# Orthotopic Glioblastoma Models for Evaluation of the Clinical Target Volume Concept

**DOI:** 10.3390/cancers14194559

**Published:** 2022-09-20

**Authors:** Rebecca Bütof, Pia Hönscheid, Rozina Aktar, Christian Sperling, Falk Tillner, Treewut Rassamegevanon, Antje Dietrich, Matthias Meinhardt, Daniela Aust, Mechthild Krause, Esther G. C. Troost

**Affiliations:** 1 OncoRay—National Center for Radiation Research in Oncology, Faculty of Medicine and University Hospital Carl Gustav Carus, Technische Universität Dresden, Helmholtz-Zentrum Dresden—Rossendorf, 01307 Dresden, Germany; 2 Department of Radiotherapy and Radiation Oncology, Faculty of Medicine and University Hospital Carl Gustav Carus, Technische Universität Dresden, 01307 Dresden, Germany; 3 National Center for Tumor Diseases (NCT), Partner Site Dresden, Germany: German Cancer Research Center (DKFZ), Heidelberg, Germany; Faculty of Medicine and University Hospital Carl Gustav Carus, Technische Universität Dresden, Dresden, Germany, and Helmholtz Association/Helmholtz-Zentrum Dresden—Rossendorf (HZDR), 01307 Dresden, Germany; 4 Helmholtz-Zentrum Dresden—Rossendorf, Institute of Radiooncology—OncoRay, 01307 Dresden, Germany; 5 German Cancer Consortium (DKTK), Partner Site Dresden, and German Cancer Research Center (DKFZ), 69120 Heidelberg, Germany; 6 Institute of Pathology, University Hospital Carl Gustav Carus (UKD), Technische Universität Dresden, 01307 Dresden, Germany

**Keywords:** glioblastoma, CTV, orthotopic model, MALDI, invasion

## Abstract

**Simple Summary:**

The accurate and precise definition of the target volume is of enormous importance for the treatment success of radiotherapy. In glioblastoma, the microscopic tumor extension is unclear, which limits the specificity of irradiation leading to either increased risk of local failure or enhanced toxicity rates. In this study, we investigated the microscopic tumor extensions of two different untreated and irradiated orthotopic brain tumor models and correlated this with histologically stained cancer stem cell markers as well as invasion markers and analyses using Matrix-Assisted Laser Desorption/Ionization (MALDI). We found specific MALDI peaks as potential markers for normal brain tissue but also others for demarcation of tumor areas. Furthermore, MMP14 staining revealed mainly positive cells in the tumor border, which could reflect the invasive front in both models. Altogether, the results of this study indicate that an individualized target volume definition for radiotherapy based on biological tumor characteristics in glioblastoma models seems possible.

**Abstract:**

In times of high-precision radiotherapy, the accurate and precise definition of the primary tumor localization and its microscopic spread is of enormous importance. In glioblastoma, the microscopic tumor extension is uncertain and, therefore, population-based margins for Clinical Target Volume (CTV) definition are clinically used, which could either be too small—leading to increased risk of loco-regional recurrences—or too large, thus, enhancing the probability of normal tissue toxicity. Therefore, the aim of this project is to investigate an individualized definition of the CTV in preclinical glioblastoma models based on specific biological tumor characteristics. The microscopic tumor extensions of two different orthotopic brain tumor models (U87MG_mCherry; G7_mCherry) were evaluated before and during fractionated radiotherapy and correlated with corresponding histological data. Representative tumor slices were analyzed using Matrix-Assisted Laser Desorption/Ionization (MALDI) and stained for putative stem-like cell markers as well as invasion markers. The edges of the tumor are clearly shown by the MALDI segmentation via unsupervised clustering of mass spectra and are consistent with the histologically defined border in H&E staining in both models. MALDI component analysis identified specific peaks as potential markers for normal brain tissue (e.g., 1339 *m*/*z*), whereas other peaks demarcated the tumors very well (e.g., 1562 *m*/*z* for U87MG_mCherry) irrespective of treatment. MMP14 staining revealed only a few positive cells, mainly in the tumor border, which could reflect the invasive front in both models. The results of this study indicate that MALDI information correlates with microscopic tumor spread in glioblastoma models. Therefore, an individualized CTV definition based on biological tumor characteristics seems possible, whereby the visualization of tumor volume and protein heterogeneity can be potentially used to define radiotherapy-sensitive and resistant areas.

## 1. Introduction

In times of high-precision radiotherapy, the accurate and precise definition of the primary tumor localization and its microscopic spread is of enormous importance for the treatment outcome of each individual patient [1]. A published review on histopathological studies of different tumor entities underlines the challenges in defining Clinical Target Volume (CTV), supporting the need for further investigations [2].

Glioma is the most commonly diagnosed primary brain tumor and, due to its malignancy, is highly correlated with a poor prognosis, especially in IDH-wildtype glioblastoma WHO grade 4 tumors. In glioblastoma, the microscopic tumor extension is uncertain, and therefore, large safety margins are currently used to derive the CTV, including the primary tumor and its microscopic spread, for radiation treatment planning. In National Comprehensive Cancer Network (NCCN) guidelines, a CTV margin of 2–2.5 cm for high-grade glioma in terms of volumetric expansion of the Gross Tumor Volume (GTV) is recommended [3]. This population-based CTV could either be too small (leading to increased risk of loco-regional recurrences) or too large (enhancing the probability of normal tissue toxicity). Clinical data demonstrated that more than 80% of glioblastoma recurrences occur within a 1–2 cm margin around the contrast-enhanced lesion on Magnetic Resonance Imaging (MRI) [3]. Nevertheless, previously published autopsy studies in patients with untreated glioblastoma showed a significant peripheral infiltration also beyond 2 cm of the contrast-enhancing tumor in Computed Tomography (CT) images [4]. Two biopsy-based studies revealed glioma cancer cells in normal brain tissue up to 4 cm away from the visible tumor edge [5,6], which further underlines the high potential of widespread tumor invasion along white matter tracts. The first approaches using automatic methods and deep convolutional neural networks for improving CTV definition are still in silico studies [7]. 

Altogether, these limited clinical data do not yet provide precise evidence for individualized CTV delineation in glioblastoma patients. Therefore, the aim of this project was to derive an individualized definition of the CTV in preclinical glioblastoma models based on specific biological tumor characteristics and image analyses. For this purpose, the microscopic tumor extensions of two orthotopic brain tumor models with different invasiveness were evaluated before and during fractionated radiotherapy and correlated with corresponding histological data. Representative tumor slices were analyzed by using Matrix-Assisted Laser Desorption/Ionization (MALDI) and stained for H&E as well as putative stem-like cell markers and invasion markers (Nestin, MMP14, Musashi 1, CD44) in order to establish the basis for a biologically-derived CTV definition. 

## 2. Materials and Methods

### 2.1. Cell Culture

G7_mCherry cells were obtained from Prof. A. Chalmers, Institute of Cancer Sciences, University of Glasgow, Scotland, UK, and maintained in stem cell culture condition. For this purpose, matrigel-coated cell culture flasks were used as well as Advanced DMEM/F12 (Invitrogen 12634028, Waltham, MA, USA) medium. Moreover, cell culture media was supplemented with 20 ng/mL Human EGF (Invitrogen PHG0313, Waltham, MA, USA), 10 ng/mL Human FGF (Invitrogen PHG0263, Waltham, MA, USA), 1% B-27 supplement (Invitrogen 17504-044, Waltham, MA, USA), 0.5% N-2 supplement (Invitrogen 17502-048, Waltham, MA, USA), 5 µg/mL Heparin (Sigma Aldrich H3393-10KU, St. Louis, MO, USA) and 1% L-glutamine (Glutamax^®^; Gibco 35050-038). Cell lines were sub-cultured 1–2 times per week, depending on the confluency. In short, cell culture media was aspirated and washed with 10 mL of PBS. Then, 5 mL accutase was added to detach cells from the surface, neutralized afterward with 10 mL of culture media and finally, cells were collected by centrifugation at 300× *g* for 5 min at 4 °C. 

U87MG_mCherry were obtained from Prof. L. Kunz-Schughart, OncoRay—National Center for Radiation Research in Oncology, Dresden, Germany, and maintained in modified Eagle’s medium (Biochrom GmbH, Berlin, Germany). The medium was supplemented with 10% fetal calf serum (Sigma-Aldrich Chemical GmbH, St. Louis, MO, USA) and 1% penicillin–streptomycin (Biochrom GmbH, Berlin, Germany). Preparation was prepared as described above, but trypsin was used instead of accutase to detach the cells from the surface.

### 2.2. Animals

Male and female NMRI (nu/nu) mice used for the experiments were obtained from the OncoRay pathogen-free animal breeding facility (Faculty of Medicine Carl Gustav Carus, Technische Universität Dresden, Dresden, Germany) and also maintained there with a constant daily cycle of 12 h light and 12 h darkness at 26 °C room temperature; fed with a commercially available laboratory mice diet (Ssniff special diets GmbH, Soest, Germany) and sterile filtered water ad libitum.

The animal facility and the conducted experiments were approved according to the German animal welfare regulations and institutional guidelines (TVV 31/2018; DD24.1-5131/449/33).

### 2.3. Initiation of Orthotopic Glioblastoma Mouse Models

All animals underwent total body irradiation while immobilized in a plexiglass tube 2–5 days prior to tumor transplantation with 4 Gy (Maxishot 200 Y.TU/320-D03, Yxlon International; 200 kV, 0.5 mm additional copper filtration, 1 Gy/min) to further reduce the residual immune system of the athymic nude mice. The antibiotic Enrofloxacin (0.25 mg/mL) was added to the drinking water for 5 days to prevent infections.

Two different glioblastoma cell lines (U87MG_mCherry, *n* = 24; G7_mCherry, *n* = 15) were transplanted orthotopically into the brain of 7 to 12 weeks old male and female immunodeficient nude mice (NMRI nu/nu) using a stereotactic technique. For the transplantation, mice were anesthetized using 16 mg/kg body weight (bw) xylazine (Rompun^®^, Bayer Healthcare) and 120 mg/kg bw ketamine (Ketamin 500 Curamed^®^, CuraMed Pharma). In total, 2.5 × 10⁵ U87_mCherry and 2 × 10⁵ G7_mCherry cells in 3 μL PBS were orthotopically transplanted with the help of a Hamilton syringe into the right hemisphere (2 mm right and 2 mm dorsal of the bregma) of mice brains using a stereotactic frame system (Stoelting Co., Wood Dale, IL, USA). After transplantation, the syringe was withdrawn slowly; the animal was removed from the frame and kept on a preheated pad until recovery. Animals with deteriorated condition were euthanized in accordance with German animal welfare regulations. 

### 2.4. MR Imaging

Tumor growth was monitored using magnetic resonance imaging (MRI) with a frequency depending on the tumor model and the corresponding growth rate (e.g., weekly imaging for U87_mCherry tumors and at least every two weeks for G7_mCherry). MRI examinations were acquired with a 1.0 Tesla nanoScan^®^ PET/MRI system (Mediso Medical Imaging Systems, Budapest, Hungary) using the mouse head coil. For this purpose, Isoflurane (2–2.5% in oxygen; Baxter Germany) was used for anesthesia, and mice were positioned in the MRI bed with an integrated warming system. Bed temperature (37 °C), as well as breathing frequency, were monitored during the whole imaging procedure. 

First, a T2-weighted 3D fast spin echo sequence with a field of view (FOV) covering the head of the mouse was performed (repetition time (TR): 1000 ms, effective echo time (TE): 97.7 ms, FOV: 31.3 mm, slice thickness: 0.23 mm, number of slices: 90). Second, a 3D gradient echo spoiled T1-weighted sequence was applied 10 min after i.p. injection of 5 mL/kg bw Omniscan^®^ (GE Healthcare, Chicago, IL, USA) Gadolinium-based contrast agent at the same position (TR: 15 ms, TE: 3.1 ms, flip angle: 25°, FOV: 60 mm, slice thickness: 0.23 mm, number of slices: 90). Data were analyzed using the InterviewFusion™ software (Mediso Medical Imaging Systems, Budapest, Hungary); the investigator was blinded to the therapy at the moment of evaluation.

### 2.5. Image-Guided Orthotopic Irradiation

After reaching a tumor diameter of 3 mm in MRI, mice were randomly assigned to the untreated control group or received fractionated irradiation (3 or 6 fractions of 3 Gy) using the Small Animal Image-Guided Radiation Therapy platform (SAIGRT) [8] and the treatment planning software µ-RayStation 8 (RaySearch Laboratories AB, Stockholm, Sweden).

Animals were irradiated with a workflow adapted from clinical routine practice: a treatment planning cone-beam CT (CBCT) as well as T1- and T2-weighted MRI scans were acquired 1–3 days prior to the first fraction for each individual mouse. Both imaging modalities were imported into µ-RayStation8 and rigidly co-registered for treatment planning. The GTV was manually contoured based on the corresponding treatment planning MRI scan, and the target isocenter position was defined as mass center of the GTV. One individual treatment plan was created for each animal using the software-assisted algorithm and equal-weighted beams at gantry angles of 0° and 270° through 3.5–5 mm diameter collimators. Dose distribution was calculated and normalized to 3 Gy per fraction average dose of the GTV.

In order to align the animal stage positioner to the target isocentre, orthogonal conventional X-ray images were obtained before every fraction and compared with corresponding digitally reconstructed radiographs from the planning CBCT to calculate the respective correction offset for positioning. Fractionated irradiation (3 or 6 fractions of 3 Gy) was applied on consecutive days, excluding weekends, with a beam quality of 200 kV (0.5 mm additional copper filtration) at a dose rate of approximately 1 Gy/min. The whole brain was excised 72 h after the last irradiation or after reaching the entering diameter in the control group for further analysis.

### 2.6. Histology

Brains of mice were fixed overnight in 4% formalin and embedded in paraffin (FFPE) for histological analysis. First, hematoxylin and eosin (H&E) staining and tumor assessment by pathologists were conducted to histologically confirm the presence and location of viable tumor cells in our orthotopic models. Second, the FFPE tissue was further processed for immunohistochemistry (IHC) under standardized conditions for investigations of putative stem-like cell markers and potential invasion markers.

Briefly, for evaluation of Ki67 staining to identify proliferating cells of human origin, the polyclonal rabbit anti-human Ki67 antibody (dilution 1:5000; abcam 833) was used. Analysis of CD44 was conducted by the monoclonal rabbit anti-human CD44 antibody (dilution 1:5000; abcam 216647). Musashi 1 was identified by a polyclonal rabbit antibody (dilution 1:100; abcam 52865). MMP14 as potential invasion marker was stained with the polyclonal rabbit anti-human MMP14 antibody (dilution 1:1000; abcam 3644), and Nestin was evaluated using the monoclonal rabbit antibody (dilution 1:10,000; abcam 176571). All stainings were processed with the Envision+ α-rabbit kit (Dako) according to the manufacturer’s instructions. 

Staining intensity was scored in blinded samples by independent observers (MM; PH and RB) for all immunohistochemical analyses and correlated with location-dependent MALDI data.

### 2.7. Mass Spectrometry (MALDI) Imaging (MSI)

Representative tumor slices were analyzed by using Matrix-Assisted Laser Desorption/Ionization (MALDI) mass spectrometry. Indium Tin Oxide (ITO)-coated glass slides were covered with Nonidet P 40 (dilution 1:1000) and Poly-L-Lysine in water (dilution 1:1). FFPE tumor tissue sections (2 µm) on ITO slides were deparaffinized following twice xylene, isopropanol, 100% ethanol, 96% ethanol, 70% ethanol and water for 5 min each. To unmask the binding sites, slides have been incubated at 110 °C, 6 bar for 20 min in HPLC water (Zytomed). Subsequently, ITO slides were dried in the vacuum device for 30 min. Sixteen cross-layers of sequencing grade porcine trypsin with 20 mM ammonium bicarbonate were sprayed on the tissue section with 10 psi nitrogen gas, 0.015 mL/min at 30 °C by HTX sprayer (HTX Technologies LLC, Chapel Hill, NC, USA) followed by enzymatic digestion inside a wet camber at 50 °C for 2 h. HCCA (200 mg) was solved in 14 mL acetonitrile and 6 mL distilled water was mixed with 200 µL TFA, and four matrix layers were sprayed on the section by HTX sprayhood (HTX Technologies, Chapel Hill, NC, USA). To calibrate mass sizes, an external peptide mixture (Peptide Calibration Standard II, Bruker Corporation, Billerica, MA, USA) was added to the matrix layers. Samples were measured by RapiFlex Tissuetyper (Bruker Corporation, Billerica, MA, USA) for 600–3200 *m*/*z* with positive ion mode and reflector detector with 1.25 Gs/s digitizer detection rate. Laser spots of five times 11 µm × 11 µm resulting in pixel sizes of 50 µm × 50 µm, were set with 500 laser shots on a frequency of 5000 Hz for application. For spatial localization of the measurement, MALDI glass slides were scanned before trypsin digestion and matrix covering. The digital slides were overlaid with the instrument spatial settings by using FlexImaging software (Bruker Corporation, Billerica, MA, USA). MALDI imaging runs were performed using FlexControl software (Bruker Corporation, Billerica, MA, USA) with standardized settings, as described for all samples. 

The resulting data sets were transferred to SCiLS Lab 2016b software (Bruker Corporation) and analyzed with MALDI imaging standard biostatistical tools (PCA, ROC, spatial segmentation, classification). Single or multiple mass peaks can be visualized back to tissue compartments and show divergent intensities (tumor-specific masses determined by mass spectra according to time-of-flight masses, given in mass-to-charge ratio [*m*/*z*]). 

## 3. Results

**Figure 1:**
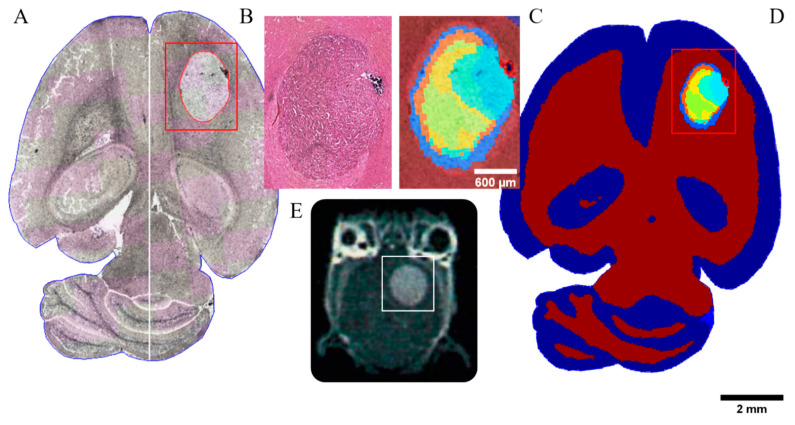
Multimodal analysis of the CTV in a representative untreated brain tumor section of a U87MG mCherry sample. (**A**) MALDI mass spectrometry image as technique for proteomics analysis measured without staining. GTV is contoured in red within the evaluated region (red box). (**B**) H&E staining of tumor region. (**C**) The edges of the tumor are clearly shown by the segmentation of the protein/peptide detection and analysis determined by means of mass spectra. The resulting mass spectrometry data are consistent with the histologic annotation of the tumor. The clustering of the spectra shows a ring-shaped separation. (**D**) Unsupervised clustering map of mass spectra. (**E**) Corresponding T1-weighted MR image highlighting the tumor area (white box).

The U87MG_mCherry cohort showed a take rate of 100% (*n* = 24), divided into 13 controls and 11 irradiated tumors, all treated with three fractions. Using MALDI mass spectrometry, tissue sections of murine brain containing tumors displayed a heterogeneous distribution of protein and peptide masses, which were used to differentiate tumor area vs. normal brain tissue. The boundaries of the tumor were clearly shown by the segmentation via unsupervised clustering of mass spectra and were consistent with the histologically defined border in H&E staining (Figure 1). The separation by clustering of the spectra has a ring-shaped appearance. Table S1 gives an overview of corresponding discriminative MSI *m*/*z* values, including mean intensity and Receiver Operating Characteristic (ROC) values of all U87MG_mCherry tumors. 

**Figure 2:**
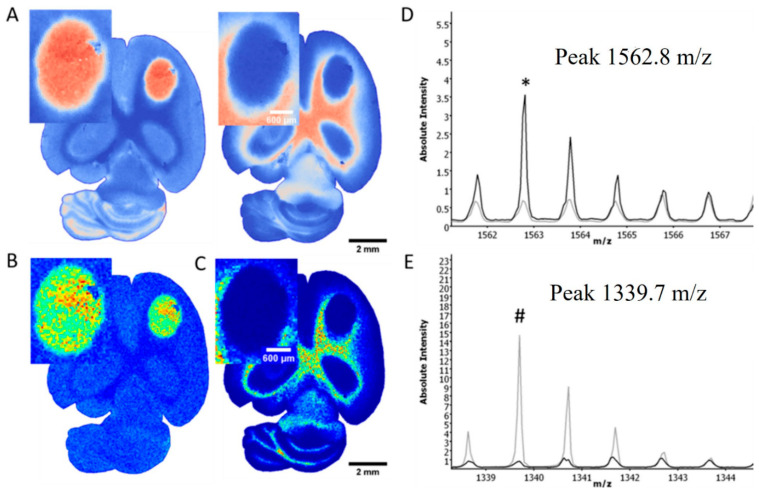
Distinguished mass information in one exemplary sample of untreated U87MG-mCherry tumor and normal brain tissue. Principle component analysis of mass spectra demonstrates clusters of similarities and distinctions based on their measurement characteristics, showing a clear separation of tumor and brain tissue (**A**). Tissue-dependent mass spectra show differences in mass distribution of both tumor (**B**) and normal tissues (**C**), e.g., mass 1562.8 *m/z* as tumor peak ((**D**); asterisk *), or mass 1339.7 *m/z* as marker for normal tissue ((**E**); octothorpe #). The black line (**D**,**E**) represents mass spectra of the whole tumor area, whereas the grey line represents mass spectra of the normal brain tissue in the same content area.

**Figure 3:**
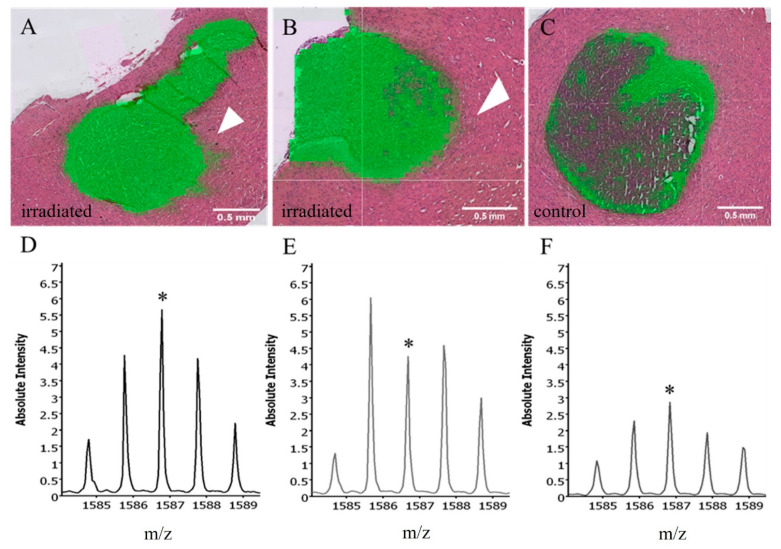
Intensity maps and blots of mass 1586.8 *m/z* (upper part: green; lower part: marked with *) in three representative U87MG_mCherry tumors indicate an extension and increase of the mass beyond the tumor margins after irradiation with 3 fractions of 3 Gy within the irradiated volume (arrows, (**A**/**D**) + (**B**/**E**)), in contrast, no expansion can be seen in untreated tumors (**C**/**F**).

**Figure 4:**
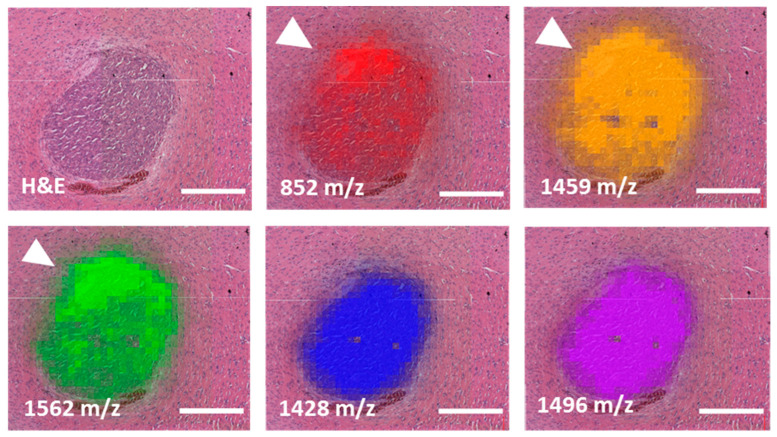
Different mass peak intensities in one representative U87MG_mCherry tissue section. Invasive front of tumor cells displayed by tumor-specific masses 852, 1459 and 1562 *m/z* and marked by an arrow showing spreading of tumor cells toward normal tissue. In contrast, two additional tumor masses, 1428 and 1496 *m/z,* did not show tumor overlapping intensities. Scale bars represent 0.5 mm.

MALDI component analysis of five untreated and five low-dose irradiated specimens (3 × 3 Gy) supported a peak of 1339 *m*/*z* as a potential marker for normal brain tissue, whereas the peak of 1562 *m/z* demarcated the tumors very well irrespective of treatment group. Figure 2 shows distinguished single mass spectra information in one exemplary U87MG_mCherry tumor and corresponding normal brain tissue measured in the same content area with both calculated areas at the same size. Using the Mascot database, a search for peak 1562 *m*/*z* identified the following human proteins: Negative regulator of P-body association (NBDY_Human), X antigen family member 1 (XAGE1_Human), Translocase of inner mitochondrial membrane 8 homolog B (TIM8B_Human), Ubiquinol-cytochrome c reductase complex III subunit VII (QCR8_Human). Comparison of untreated and irradiated tumors revealed differences regarding the tumor border. Figure 3 indicates, for example, an extension of the mass 1586.8 *m*/*z* beyond the tumor margins after irradiation within the irradiated area, whereas no expansion can be seen in untreated tumors. The same observations can be made by evaluating different mass peak intensities in one representative U87MG_mCherry section (Figure 4). Specific intensity maps of single peaks (e.g., 852, 1459 and 1562 *m*/*z*) simultaneously represent tumor area and possible invasive front of tumor cells spreading toward normal tissue. 

**Figure 5:**
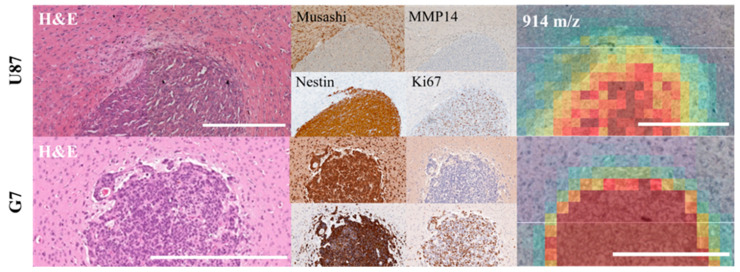
IHC staining in one representative tumor of both models (same markers for upper row: U87MG_mCherry, lower row: G7_mCherry, respectively). H&E, Musashi 1, MMP14, Nestin and Ki67 staining and MALDI mass intensity map (914 *m/z*) of the same tumor region. Intensity relations of spectra are analogous with, e.g., MMP14-rich cell areas in U87MG-mCherry. Scale bars represent 0.5 mm.

Histologically, positivity of Nestin and CD44 was not limited to a small subset of cells but was more widespread in the U87MG_mCherry model in both treatment groups (Figure 5). In contrast, Musashi 1 and MMP14 staining revealed only a few positive cells, mainly in the tumor border, which could possibly reflect an invasion zone. Spatial correlation of mass intensity maps with IHC staining showed intensity relations of 914 *m/z*, as one representative mass, that were similar with, e.g., MMP14-rich cell areas (Figure 5). 

For G7_mCherry, the take rate was 93% (*n* = 15), randomized into 5 untreated controls and 10 irradiated tumors (5 × 3 fractions and 5 × 6 fractions). As already shown for the U87MG_mCherry cohort, a clear distinction between the malignant tumor area and surrounding normal brain tissue using MALDI imaging was also possible in this tumor model. Nevertheless, several tumor peaks were also present in normal brain tissue since the G7_mCherry tumor cells are much more invasive compared to U87MG_mCherry. Figure S1 displayed the most relevant tumor-specific masses for one representative orthotopic G7_mCherry tumor. In contrast to the first tumor model, a more infiltrating growth pattern was visible via unsupervised clustering of mass spectra without additional staining. An overview of discriminative MSI *m/z* values, including mean intensity and ROC value of the entire cohort, are provided in Table S2. 

Corresponding IHC staining clearly shows missing distinct tumor borders in contrast to U87MG_mCherry (Figure 5). Again, Nestin and CD44 were widespread within the tumors independent of treatment group. In contrast, MMP14 staining was re-confirmed to be limited to a small subset of cells, possibly reflecting the invasion front.

**Figure 6:**
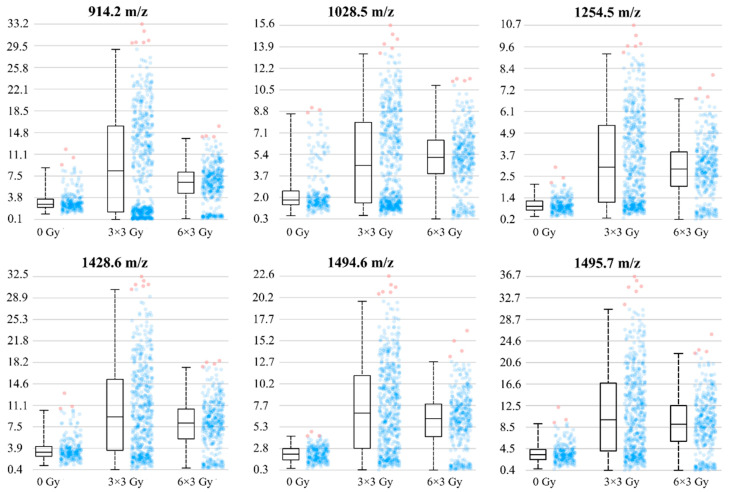
The intensity blots of tumor-specific masses are presented for differently treated G7_mCherry orthotopic brain tumors. Intensities of tumor masses increased after irradiation with three fractions of 3 Gy, suggesting a more pronounced invasion front after irradiation. Higher radiation doses of six fractions of 3 Gy did not enhance those effects. After irradiation, a broader range of intensities was shown for tumor measurement spots (dots displayed on the right of the box), suggesting a higher range of intratumoral heterogeneity of mass occurrence.

To quantify the effect of irradiation on this area, mass intensity blots are presented for differently treated G7_mCherry orthotopic brain tumors (Figure 6). After low dose irradiation (three fractions with 3 Gy), the intensities of tumor-specific masses increased, suggesting a more pronounced invasion front. As also shown in Figure 6, higher irradiation doses of six fractions with 3 Gy did not enhance those effects. In general, after irradiation, a broader range of intensities was shown for tumor measurement spots, suggesting a higher range of intratumoral heterogeneity of mass occurrence in these cases. 

## 4. Discussion

In our study, the borders of two different orthotopic brain tumor models (U87MG_mCherry; G7_mCherry) were clearly defined by the MALDI segmentation via unsupervised clustering of mass spectra before and during fractionated radiotherapy. These findings were consistent with the histologically defined tumor boundaries in H&E stainings of both models. Furthermore, MALDI component analysis showed specific peaks as potential markers for normal brain tissue, whereas other peaks demarcated the tumors very well, irrespective of the treatment group.

Since MALDI imaging has been introduced as a unique technique for proteomics analysis, it could be the basis for the identification of a new era of biomarkers [9]. It combines the protein/peptide detection and analysis determined by means of mass spectra and the macroscopic anatomy of the visible, morphological shape of tissue compartments. So far, limited data exist on the usability of this method in irradiated tissue, both in clinical application and preclinical experiments. One representative study focused on the irradiation of normal brain and intestine tissue in mice and found proteomic links to radiation response markers [10]. Therefore, the authors suggested a possible use for the prediction of unusual side effects. Another preclinical experiment investigated differences in protein spectra in irradiated brain tumor tissue and found 77 peaks with significant changes. Wibom and colleagues [11] concluded that this could help to further understand the biological effects of irradiation. These reported differences in MALDI peaks after irradiation are in line with our results. Compared to controls, the invasion front was more pronounced in both tumor models after low-dose irradiation (three fractions with 3 Gy). Higher irradiation doses of six fractions with 3 Gy applied in the G7_mCherry cohort did not enhance those effects. On the one hand, the importance of specific tumor niches, such as the invasion front, became increasingly clear over the past years. On the other hand, the effects of radiotherapy and the impact of invasion front on stem-like cells have been controversially discussed. One hypothesis suggests that stem-like cancer cells might be dominantly situated in the invasion front, as cancer invasion and metastatic spread should be initiated mainly from this specific part of tumors. If this is true, the number and individual radiosensitivity of these stem-like cancer cells would significantly influence the necessary dose for a cure and the margins of the target volume [12]. Radiotherapy-induced invasiveness after single-dose irradiation has also been reported by Wang and colleagues [13], showing mobilization of macrophages and tumor revascularization as potential underlying mechanisms. Specific mass peaks seem to correlate with these niches and could be the basis for target characterization.

In general, MALDI data seem to have enormous potential for different clinical applications: one conceivable aspect would be visualization of tumor volume and protein heterogeneity for the definition of potentially radiotherapy-sensitive and resistant areas. Mass spectrometry is clinically possible in either biopsy material or sections of resected specimens and could also be used to identify specific proteins as additional biomarkers for individualized therapeutic approaches; a proteomic comparison of glioblastoma samples with normal tissue measurements revealed twenty-two attractive molecular targets, e.g., for immunotherapy, which could overcome therapy resistance and possibly improve survival of this patient cohort [14]. Since overexpression of Epidermal Growth Factor Receptor (EGFR) is common in many glioblastoma patients, it could also be considered a potential target. Nevertheless, the first clinical trials investigating EGFR inhibition in recurrent glioblastoma provided insufficient success, e.g., [15,16]. Randall and colleagues [17] used MALDI imaging for a quantitative map distribution of EGFR inhibitor and tumor characteristics in a patient-derived xenograft model, which could possibly help to analyze the reasons for drug resistance. This could be a basis for promising clinical applications of individualized drug schedules. 

Since technical improvements have led to the era of high-precision radiotherapy, the accurate definition of the target volumes is of enormous importance for the treatment outcome of each individual patient [1]. Especially in glioblastoma, the microscopic tumor extension is uncertain, and therefore, large uniform safety margins are currently being used to define the CTV for radiotherapy [3,18]. The first investigations for biologically-driven individualization of target volumes for glioblastoma patients focused on MGMT status—in two studies, MGMT methylation was significantly correlated with more out-of-field recurrences [6,19]. Therefore, the authors concluded that MGMT status could be associated with the degree of microscopic extension. For low-grade glioma, data exist regarding patterns of failure in IDH mutated patients, showing that not all recurrences occur within a 5 mm margin of the GTV [20]. Putative stem-like cell markers, as well as invasion markers, are also possible factors for the establishment of a biologically individualized CTV. In particular, in the U87MG_mCherry cohort, Musashi 1 and MMP14 staining revealed only a few positive cells, mainly in the tumor border. These areas could reflect an invasion zone and, consequently, the possible need for corresponding CTV extension. Further preclinical experiments in glioblastoma revealed that dual targeting of integrin and JNK pathways led to a strong and significant reduction of the invasion capacity of stem-like cells [21]. Additionally, matrix metalloproteinase markers have already been shown to correlate with tumor invasiveness in patients with head and neck squamous cell carcinoma [22], which is also in line with our current data. 

To strengthen the translational potential of our results, the correlation of MALDI data and biomarker staining to clinically used imaging techniques should be investigated. The question is whether intratumoral heterogeneity of the proteome can be linked to MRI or other imaging modalities in order to use them for target volume definition of radiotherapy. In a recently published post-mortem study on human brain tissue, regional lipid abnormalities correlated well with MRI-defined white matter changes [23]. Other innovative approaches investigated methods for the integration of advanced imaging techniques (e.g., imaging mass cytometry or in vivo MRI) with MALDI data [24,25]. If clinically used sequences could predict proteomic information, it could open a wide range of new possibilities. Therefore, to further improve our CTV model, MRI data will be analyzed using radiomics or more sophisticated analyses in a next step. Afterward, a validation experiment is needed before translating this preclinically established model into a clinical study.

## 5. Conclusions

The results of this study indicate that MALDI information correlates with microscopic tumor spread in glioblastoma models. Therefore, individualized CTV definition based on biological tumor characteristics seems possible, whereby visualization of tumor volume and protein heterogeneity can be potentially used to define radiotherapy-sensitive and resistant areas. 

## Data Availability

The data presented in this study are available on request from the corresponding author.

## Supplementary Materials

The following supporting information can be downloaded at: https://www.mdpi.com/article/10.3390/cancers14194559/s1, Figure S1: MALDI imaging of one exemplary G7_mCherry tumour. Most relevant tumour specific masses are displayed. No clear tumour border can be visualized, Table S1: List of discriminative mass values (*m/z*) for U87MG_mCherry tumours including mean intensities and ROC values of background vs. tumour and tumour vs. background analysis, respectively. Table S2: List of discriminative mass values (*m/z*) including mean intensities and ROC values of tumours. Several tumour peaks were also present in normal brain tissue since the G7_mCherry tumour cells are much more invasive compared to U87MG mCherry.
