# Supplementary material for: Orthotopic Glioblastoma Models for Evaluation of the Clinical Target Volume Concept
Source: Cancers (Basel). 2022 Sep 20;14(19):4559. doi: 10.3390/cancers14194559 (PMC9559695; doi:10.3390/cancers14194559)
Supplement: Supplementary file 1 [file cancers-14-04559-s001.zip › cancers-1873491-supplementary.pdf]

Supplementary Materials:

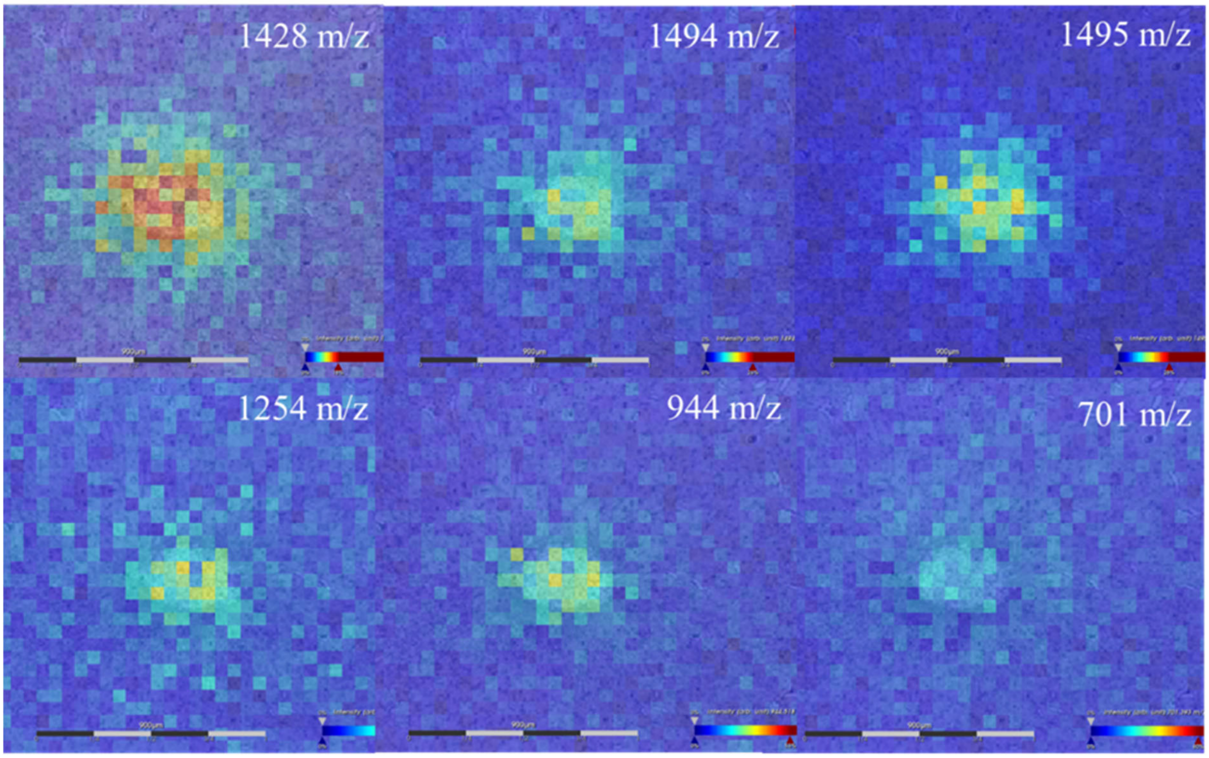

Figure S1. MALDI imaging of one exemplary G7_mCherry tumor. Most relevant tumor specific masses are displayed. No clear tumor border can be visualized.

Table S1. List of discriminative mass values (*m*/*z*) for U87MG_mCherry tumors including mean intensities and ROC values of background vs. tumor and tumor vs. background analysis, respectively.

Mass Value (*m*/*z*)	Mean Intensity (200 mDa)	AUC	Distribution
615.3	10.84	1	Brain tissue
643.3	39.45	1	Brain tissue
705.4	29.28	1	Brain tissue
707.4	6.04	1	Brain tissue
722.4	4.46	1	Brain tissue
836.4	2.96	0.999	Tumor
852.5	3.39	0.997	Tumor
898.5	2.64	0.996	Tumor
914.5	2.92	0.998	Tumor
915.5	3.06	0.996	Tumor
943.6	3.08	0.986	Tumor
944.5	10.12	0.987	Tumor
1028.6	3.12	0.993	Tumor
1095.6	2.04	0.996	Tumor
1106.6	2.23	0.999	Tumor
1339.7	11.17	0,999	Brain tissue
1428.7	3.02	0.999	Tumor
1428.7	1.13	0.999	Tumor
1429.7	1.12	0.999	Tumor
1459.7	0.91	0.996	Tumor
1465.7	1.03	0.996	Tumor
1494.7	1.48	0.997	Tumor
1495.8	1.71	0.999	Tumor
1496.8	1.26	0.999	Tumor
1562.8	0.5	0.994	Tumor

Table S2. List of discriminative mass values (m/z) including mean intensities and ROC values of tumors. Several tumor peaks were also present in normal brain tissue since the G7_mCherry tumor cells are much more invasive compared to U87MG mCherry.

Mass value (m/z)	Mean intensity (150 mDa)	AUC	Distribution
914.3	1.886	0.595	Tumor / Brain tissue
1028.5	1.459	0.689	Tumor / Brain tissue
1254.6	0.732	0.600	Tumor / Brain tissue
1428.7	1.153	0.907	Tumor
1429.6	1.036	0.671	Tumor / Brain tissue
1430.7	0.672	0.664	Tumor / Brain tissue
1494.7	1.025	0.587	Tumor / Brain tissue
1495.8	1.325	0.737	Tumor / Brain tissue
1496.1	0.955	0.743	Tumor / Brain tissue
1497.8	0.621	0.727	Tumor / Brain tissue
